# Conception and Development of the Warmth/Affection Coding System (WACS): A Novel Hybrid Behavioral Observational Tool for Assessing Parent-to-Child Warmth

**DOI:** 10.1007/s10802-023-01055-y

**Published:** 2023-04-20

**Authors:** Ashneeta H. Prasad, Yvette Keevers, Silvana Kaouar, Eva R. Kimonis

**Affiliations:** School of Psychology, The University of New South Wales, 2052 Sydney, NSW Australia

**Keywords:** Warmth, Affection, Callous-unemotional traits, Behavioral observation, Parenting, Observational coding

## Abstract

A vast body of research and theory underscores the importance of parental warmth/affection (hereby ‘warmth’ and ‘warmth/affection’ are used interchangeably) as a distinct relational process that is fundamental to core developmental processes including parent-child attachment, socialization, emotion recognition and responsivity, and empathic development. The increasing focus on parental warmth as a viable transdiagnostic and specific treatment target for Callous-Unemotional (CU) traits highlights the critical need for a reliable and valid tool for measuring this construct within clinical contexts. However, existing assessment methods have limitations in their ecological validity, clinical utility, and the comprehensiveness of their coverage of core warmth subcomponents. In response to this clinical and research need, the observational Warmth/Affection Coding System (WACS) was developed to comprehensively measure parent-to-child warmth/affection. This paper chronicles the conception and development of the WACS, which adopts a hybrid approach of utilizing both microsocial and macro-observational coding methods to capture key verbal and non-verbal subcomponents of warmth that are currently underrepresented or poorly captured by existing assessment tools. Recommendations for implementation and future directions are also discussed.

**Supplementary Information:**

The online version contains supplementary material available at 10.1007/s10802-023-01055-y.

Parental warmth is a complex and multifaceted resource to children that is vital to their survival and healthy socioemotional development. Parental warmth is one of many mechanisms that humans have evolved to extract information from their environment to determine whether it is safe, and whether their caregiver can reliably meet their needs (O’Neill et al., 2021; Floyd, 2001). Various operational definitions exist for parental warmth and affection across the literature; however, general consensus is that warmth is underpinned by both verbal (e.g., “*I love you so much*”) and non-verbal (e.g., positive physical touch including hugging and caressing) affirmations of love, fondness, enthusiasm, and positive regard towards the child that signal safety, acceptance, and are responsive to their needs (Baumrind, 1967). The current report provides an overview of the importance of parental warmth for healthy socioemotional development, the subsequent emergence of warmth-focused parenting interventions, and the strengths and limitations of current tools for assessing parental warmth. Next, we chronicle the development and refinement of a novel behavioral observation coding system (Warmth/Affection Coding System; WACS), which was designed to both address limitations of existing tools and for use as a clinical tool to accompany the delivery of emerging warmth-focused interventions.

The positive impact of parental warmth and affection is evident at multiple psychophysiological and psychosocial levels. Across animal and human studies, increased maternal care and warmth is linked with better stress modulation (Rincón-Cortés & Sullivan, 2014; Letourneau et al., 2011) that can influence gene expression (Kommers et al., 2015; Peña et al., 2013). Early maternal care is also linked with positive functional changes in the neuroanatomic structure of infants and children (Lee et al., 2019; Lupien et al., 2011). Parental warmth and sensitivity are also instrumental to establishing a secure attachment style (O’Neill et al., 2021), which in turn underpins a multitude of affective and interpersonal relational patterns throughout life (Ainsworth, 1979). Positive parent-child relationships characterized by warm, responsive parenting are critical to socialization efforts (Kochanska, Forman, Aksan et al., 2005), and empathy development by providing a vehicle through which modelled prosocial behaviors are internalized and later repeated by the child (Kochanska, 2002a, b). Taken together, parental warmth is essential for supporting healthy socioemotional development in children and can be protective against developing psychopathology across the lifespan.

Conversely, low parental warmth is a risk factor for psychopathology, such as Callous-Unemotional (CU) traits. CU traits are associated with parent-child attachment problems (Kohlhoff, Mahmood et al., 2020), pervasive emotion processing deficits (Blair et al., 2014), poor socialization as reflected by chronic and aggressive antisocial behavior, and core empathic deficits (e.g., lack of remorse/guilt, callous lack of empathy) (Frick & Viding, 2009). Children with conduct problems (CP) and co-occurring CU traits (CP + CU) display a more chronic pattern of serious aggressive and antisocial behavior that places them at greater risk for several negative life outcomes relative to children with CP-only (Frick et al., 2014).

Low parental warmth is thought to give rise to CP + CU in the presence of dispositional risk factors in the child. Specifically, children with CU traits display low affiliative reward (i.e., devaluing and not deriving pleasure from close relationships), low threat sensitivity (i.e., fearless temperamental style that is punishment insensitive, reward dominant and sensation seeking), and poor interpersonal emotional sensitivity (i.e., deficient attention, recognition, and reactivity to other’s distress cues) (Waller & Wagner, 2019). These inherited difficulties are exacerbated when exposed to a parenting environment that is low in warmth, by limiting opportunities to experience critical socioemotional learning (Waller et al., 2015). Indeed, prior studies demonstrate that lower levels of parental warmth predict higher CU trait levels in high-risk preschoolers (Waller et al., 2014), and older children (Pardini et al., 2007; Frick et al., 2003). To compensate for their dispositional difficulties, children with or at risk for CU traits require consistent exposure to emotional expressions delivered via warm parenting across various contexts to learn the socialization processes that typically developing children can organically extract from their environments.

Considering the poor outcomes associated with a lack of parental warmth, interventions are increasingly incorporating warmth as a key treatment target. Notably, targeted treatments are emerging for children with CP + CU that focus on increasing parental warmth during parent-child interactions through emotional engagement training (Dadds et al., 2019), in-vivo coaching to increase parental use of verbal and non-verbal warmth and affection (Kimonis et al., 2019), or improving ‘positive communication’ within families (Kolko & Pardini, 2010), with some promising results (Kimonis et al., 2019; Kolko & Pardini, 2010). However, few of these studies have specifically examined whether parental warmth changed in response to intervention (Kimonis et al., 2019; Kolko & Pardini, 2010). To evaluate whether these targeted interventions are achieving their aims, comprehensive tools for measuring warmth are needed to assess and track changes in response to treatment.

## Current Measures of Parental Warmth

There is a proliferation of tools available for measuring parental warmth and related parent-child processes, in part due to the varied terminology used to define these constructs. Parenting practices are most frequently enacted in the home, making parent-report questionnaires a common assessment approach. There are several questionnaires available to researchers that assess broad parenting constructs related to warmth and nurturance (see Hurley et al., 2014, and Locke & Prinz, 2002 for comprehensive reviews). Despite being in copious supply, measures for assessing warmth are limited in three key ways.

First, parental warmth is inconsistently defined across measures (Lindheim & Shaffer, 2017) and critical subcomponents that are integral to warm parenting, such as non-verbal affiliative cues that foster close, enduring relational bonds (e.g., animated facial expressions, modulation of tone/pitch of voice, and eye contact) are often overlooked. Differences in operationalizations across questionnaires can weaken their content validity (Locke & Prinz, 2002), produce divergent findings, and limit a more comprehensive assessment. Second, reviews of measures for parenting constructs find that few tools report psychometrics (Holden & Edwards, 1989; Hurley et al., 2014), and of those that do, several demonstrate inadequate psychometric properties (e.g., Parenting Relationship Questionnaire; Kamphaus & Reynolds, 2006; Parent Behavior Frequency Questionnaire; Mowder, 2000; Parent Dimensions Inventory; Power, 2002), undermining the utility of these tools in clinical contexts (Hurley et al., 2014). Third, assessing parenting through self-report presents challenges, including parents tending to use broad estimates when reporting on high frequency behaviours occurring over protracted periods of time (Tourangeau et al., 2000; Morsbach & Prinz, 2006), and engaging in biased responding (Krumpal, 2013; Morsbach & Prinz, 2006). Given these limitations, alternative methods have proven useful for enabling comprehensive, psychometrically robust, and ecologically valid assessments of warmth.

Quasi-observed measures are one potential alternative to parent-report questionnaires for providing a detailed and ecologically valid assessment of parental warmth. A popular example of such a measure is the collection of Five-Minute Speech-Samples (FMSS) and coding their content using the Family Affective Attitude Rating Scale (FAARS; Bullock & Dishion, 2004; Bullock & Dishion, 2007). Parents are asked to spontaneously express their thoughts and feelings regarding their child into an audio-recorder for five minutes. Their speech-samples are later rated by trained coders on a global (i.e., Likert) scale across several constructs including both positive (e.g., warmth, expressing love/care) and negative (e.g., criticism) parent-child processes (Pasalich et al., 2011a, b). In addition to being similarly cost-effective to parent-report questionnaires (Bullock & Dishion, 2007), the FAARS demonstrates promising psychometric properties (Weston et al., 2017; Rea et al., 2020). Despite these strengths, the FAARS speech-sample method taps into parental *attitudes* and does not provide a direct, detailed assessment of warm parenting behaviors that can be used easily within clinical treatment contexts.

Arguably, the optimal method for assessing parental warmth is observational behavioral coding. Behavioral observations are integral to evidence-based assessment and serve as the most direct (i.e., less susceptible to social desirability), ecologically valid, and clinically useful method for assessing parent-child interactions and parenting quality (Hawes & Dadds, 2006; Wysocki, 2015). There are several macro-observational (or macro) coding systems that use summary ratings on Likert scales that capture global impressions of parental warmth (e.g., Family Coding System [FCS]; Margolin & Gordis, 1992; Iowa Family Interaction Rating Scales [IFIRS]; Melby et al., 1998), as well as broad, related components (e.g., “sensitive responding” in the Coding of Attachment-Related Parenting, [CARP], Matias et al., 2006; “nurturing/supportive” in the Coder Impressions Inventory, [COIMP], Dishion et al., 2004). For example, the Family Observation Schedule—6th Edition (FOS-VI; Pasalich & Dadds, 2009) is a macro coding system that can be used across semi-structured and free-play tasks to assess various parent-child processes, including a broad ‘parental warmth’ category rated on a 5-point Likert scale capturing consistency and intensity of parent behaviors. The FOS-VI ratings consider both verbal and non-verbal behaviors, and has previously demonstrated excellent inter-rater reliability (e.g., ICC = 0.86; Pasalich et al. 2011a), and clinical validity (Dadds & Hawes, 2006; Pasalich et al., 2011b). Additionally, other macro coding systems are used to assign global ratings for specific subcomponents of warmth between parent-child dyads during structured tasks (e.g., ‘I-Love-You’ protocol used to code a 90-second ‘I-Love-You’ task; Dadds et al., 2012). In this task, parents are instructed to look their child in the eye and *“…show him/her, in the way that feels most natural for you, that you love him/he*r”, which is coded on various domains (including ‘physical’ and ‘verbal’ affection) by trained researchers on a 5-point Likert scale (1 = *Not at all*, 5 = *Very much*).

Macro coding systems tend to be more efficient since they require less intensive training to establish reliability between coders (Gridley et al., 2019). However, what is gained in efficiency is lost in depth of assessment (Bank et al., 1990) as macro coding systems may not provide detailed assessments of multifaceted constructs such as parental warmth. For example, several tools operationalize parental warmth in an aggregate manner by collapsing across several categories of verbal and non-verbal indicators (e.g., FOS-VI; Pasalich & Dadds, 2009; IFIRIS, Melby et al., 1998; FCS, Margolin & Gordis, 1998). The resultant lack of detailed clinical information (e.g., frequency of different types of verbal and non-verbal warmth cues) limits the ability to test the distinct influences of specific subcomponents of warmth on child outcomes that can be useful when refining interventions. Similarly, global scores from macro coding have limited clinical utility in their ability to inform treatment planning or monitor treatment-related changes.

Alternatively, micro coding systems enable a detailed assessment of parental warmth subcomponents. Micro coding systems capture more specifically defined, discrete units of behaviors, and can be used to collect moment-to-moment information over an interval (e.g., proportions) or a continuous (e.g., frequency/tally counts) period. Examples include the Behavioral Coding System (BCS; McMahon & Estes, 1994), the Family Interaction Coding System (Patterson et al., 1969), the Family Peer Process Code (Crosby et al., 1998), and the Specific Affect Coding System (SPAFF; Coan & Gottman, 2007). Micro coding systems can capture changes during and following treatment given the granular nature of coding units (Hawes et al., 2013). A trade-off for the additional sensitivity and nuance of micro coding systems is the extra resources required to implement them. Micro coding systems typically involve more initial and ongoing training than macro approaches to ensure inter-rater reliability and minimize coder drift (Hawes et al., 2013). Micro coding systems can also be challenging to apply to non-discrete behaviors (e.g., responsiveness), or to behaviors that occur at a high frequency (e.g., eye contact), leading researchers to adopt hybrid methods.

There is growing support for hybrid coding systems because of their ability to capitalize on the advantages of both micro and macro coding approaches. An example of a hybrid coding system that has demonstrated considerable clinical utility for assessing and monitoring positive parenting practices is the Dyadic Parent-Child Interaction Coding System—Fourth Edition (DPICS-IV; Eyberg et al., 2013). The DPICS-IV is used to assess the quality of parent-child interactions across three, 5-minute play situations (child-led play, parent-led play, clean-up) following a 5-minute warm-up period. This coding system was originally developed to assist with treatment planning and tracking for families undergoing Parent-Child Interaction Therapy (PCIT; Eyberg & Funderburk, 2011), and has been iteratively refined over decades. PCIT is a parent management training program that seeks to both enhance the quality of the parent-child relationship by coaching parents to implement positive parenting practices during play situations, and to train parents in using consistent, evidence-based discipline techniques to promote child compliance. PCIT has been found to be efficacious for reducing childhood externalizing problems across numerous clinical trials (Thomas & Zimmer-Gembeck, 2007). The main DPICS-IV categories can be broadly classified into ‘Do’ and ‘Don’t’ skills that are taught to parents at the beginning of PCIT.

DPICS-IV ‘Do skills’ include delivering praise (labeled and unlabeled), reflecting speech back to the child (i.e., parroting), and describing the child’s appropriate behavior. Imitating alongside the child’s play and engaging with enthusiasm are also emphasized. DPICS-IV ‘Do skills’ constitute child-led play skills that overlap with elements of verbal warmth and are important for strengthening the parent-child relationship (Eyberg & Funderburk, 2011; Eyberg & Bussing, 2011). The frequency of parent’s use of praise, reflections and behavior descriptions are tallied, alongside their use of verbal statements that undermine the quality of the parent-child interaction (also referred to as DPICS-IV ‘Don’t skills’; e.g., questions, negative/critical talk, commands). Clinicians also assign macro ratings on a 2-point scale to indicate the parent’s level of enthusiasm (“*Satisfactory*”, “*Needs practice”*), and imitation of the child’s appropriate behavior as well as their ability to ignore any disruptive child behavior on a 3-point scale (“*Satisfactory*”, “*Needs practice*”, “*Not applicable”*). The DPICS-IV has considerable clinical utility and is a required component of the PCIT protocol that is used to guide intensive in-vivo coaching, and to determine when families are ready to progress to the second discipline phase of treatment. The DPICS-IV has consistently demonstrated good psychometric properties, including good inter-rater reliability, sensitivity to change, and correlates with other parenting criterion measures (Cotter & Brestan-Knight, 2020; Kohlhoff, Morgan et al., 2020). While the DPICS-IV is useful in clinical and research contexts for assessing primarily verbal parenting practices, it is limited in its current ability to adequately capture parental warmth for three key reasons.

First, the DPICS-IV focuses primarily on parent verbalizations governed by extensive coding rules that can risk underestimating levels of warmth. For example, parent verbalizations describing their own positive experience (e.g., “*This is a lot of fun!”*), expressing their desires, even if in relation to the child (e.g., *“I really want to draw with you!”*), or in the context of trying to attune to the child’s feelings (e.g., *child smiles widely while being hugged* “*Aww you love big cuddles”*) are coded as “Neutral Talk”. As such, positively valanced statements that add to the overall quality and warmth of the parent-child interaction are not always categorized as positive parenting behaviors under the DPICS-IV.

Second, the delivery and affective quality of verbal statements (e.g., vocal tone/pitch modulation) is not considered or quantified for positive parenting categories in the DPICS-IV. When considering the construct of warmth, *how* verbal statements are delivered can also impact on the quality of the parent-child relationship (Floyd & Ray, 2003; Feldman, 2012). The only existing reference to tone of ‘vocalization’ in the DPICS-IV is for the use of sarcastic/sassy tones or parental whining which are coded as “Negative Talk” (i.e., critical or disapproving statements). Thus, including greater coverage of positive modulation of tone and pitch of voice is important for capturing parental efforts to convey warm engagement.

Third, the DPICS-IV insufficiently captures key non-verbal affiliative behaviors that are central to fostering warmth and affection. Currently, the DPICS-IV includes limited reference to the underutilized and understudied “positive” (e.g., stroking child’s hair, high-fiving child) and “negative” touch categories. However, the attachment-rich qualities of other non-verbal behaviors (e.g., animated facial expressions, attempts to engage and sustain reciprocal play) (Dadds & Hawes, 2006; Eyberg & Funderburk, 2011) are either not measured or assessed using crude macro scales. For example, the “imitation” category utilizes a broad, 2-point scale that assesses parent efforts to engage in *similar* play *alongside* the child (e.g., constructing a tower next to child who is building), in turn, overlooking the important non-verbal cues associated with parent attempts to engage their child in and/or sustain reciprocal play *with* their child. In sum, while the DPICS-IV provides an excellent framework for assessing positive parenting practices and clinically complements PCIT, it requires extension to measure parental warmth more comprehensively.

Akin to the way in which the DPICS was developed as an accompanying tool for assessing positive parenting practices in PCIT, new adaptations of existing observational coding systems often occur in response to emerging theories or interventions that require appropriately tailored assessment tools (Hawes et al., 2013). One such intervention is PCIT adapted to target the vulnerabilities of children with CP + CU (‘PCIT-CU’; Kimonis et al., 2019; Fleming et al., 2022), including improving low parental warmth/affection to foster empathy development and emotional processing skills in these dispositionally vulnerable children. Consequently, a need has emerged for an accompanying assessment tool to measure and monitor changes across the treatment targets of PCIT-CU. PCIT-CU adapts the first phase of PCIT by explicitly in-vivo coaching parents to increase their use of verbal (e.g., vocally expressed affection, modulation of voice) and non-verbal (e.g., animated facial expressions, eye contact) displays of warmth towards their child (Fleming et al., 2022).

## Warmth/Affection Coding System (WACS—1st Edition; WACS-I)

The following section describes the development of a new, comprehensive hybrid coding system intended to be used in tandem with PCIT-CU to assess and monitor changes in parental levels of verbal and non-verbal warmth within clinical and research contexts. The WACS-I aimed to overcome limitations of existing warmth/affection measurement methods by: (i) employing a direct behavioral observational system that, (ii) utilizes a hybrid micro and macro coding approach to, (iii) comprehensively capture key verbal and non-verbal subcomponents of warmth, for (iv) use in research and clinical settings to assess and monitor treatment progress and outcomes among children aged 3 to 7. Furthermore, the WACS-I was also developed to be used in tandem with the DPICS-IV to provide more wholistic coverage of verbal and non-verbal behaviors occurring within a parent-child dyad that are critical to the quality of the relationship. The remainder of this report will describe the development of the WACS-I across two phases, as well as recommendations for its use in various settings.

### Phase 1: Video Review and Development

**Fig. 1:**
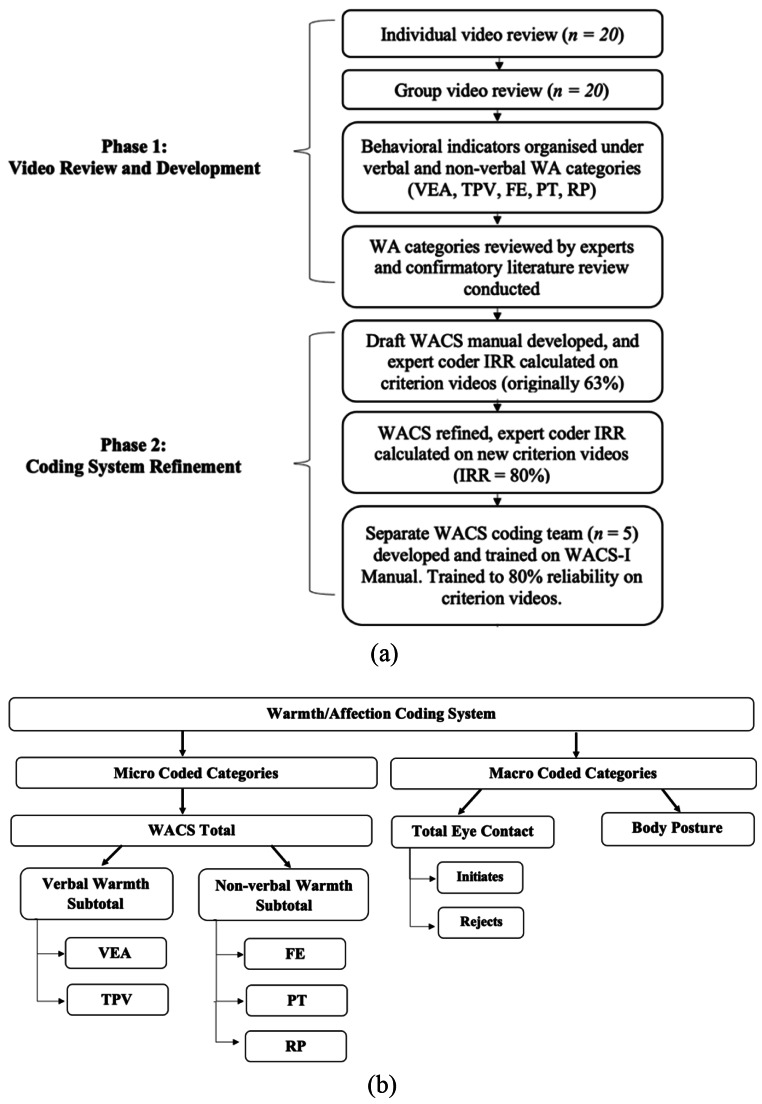
(a) Development process and (b) overview of WACS-I categories. Note: WA = Warmth/Affection; WACS = Warmth/Affection Coding System; WACS-I = Warmth/Affection Coding System − 1st Edition; VEA = Vocally Expressed Affection; TPV = Tone/Pitch of Voice; FE = Facial Expression; PT = Physical Touch; RP = Reciprocal Play

The section below outlines the video review process (see Fig. 1a) of the development team to collate and organise possible coding categories from parent-child observations, followed by a review of the empirical support for WACS-I coding categories. The development team consisted of trained clinical undergraduate and postgraduate psychology research students who both individually and collectively reviewed 20 videos of parent-child interactions (child M_age_= 4.5 years; child ethnicities included *n* = 18 [90%] Caucasian; *n* = 1 [5%] Asian; *n* = 1 [5%] other race/ethnicity) recorded as part of 15-minute DPICS-IV observation scenarios (i.e., 5-minutes each of child-led and parent-led play and clean-up) conducted during a pre-treatment assessment for clinic-referred children with CPs.

Coding categories extracted from parent-child videos were guided by a priori hypotheses informed by theoretical models that supported PCIT and the importance of warmth/affection in the parent-child relationship. These included Social Learning Theory, Attachment Theory, and wider research on both verbal and non-verbal affiliative cues (Bandura, 1965; Ainsworth, 1979; Floyd & Morman, 2001). During the video review process, parent-child behaviors thought to contribute to a warm, affectionate, and emotionally enriching interaction that were not fully captured by the DPICS-IV were identified and organized into broad coding categories. Several verbal and non-verbal indicators of warmth/affection consistently emerged across individual and group reviews of parent-child interactions that were either not currently assessed (e.g., animated facial expressions, modulation of voice tone/pitch, body posture), or not comprehensively captured by the DPICS-IV (e.g., vocally expressed affection, reciprocal play, eye contact). Panel review by experts in clinical child psychology and a confirmatory literature review were conducted to support the inclusion of these coding categories in the manual (see Fig. 1b). Findings of the literature review are detailed below.

### Empirical Support for Coding Categories

#### Vocally Expressed Affection (VEA)

VEA refers to verbal parental affirmations of love, endearment, and/or positive regard towards the child (e.g., “*I love playing with you*”, “*You are such fun to be around*”). Such statements signal approval, nurturance, and can strengthen children’s self-esteem (Khaleque & Rohner, 2002; Khaleque, 2013). VEA also fosters shared positive affect within the dyad (Kochanska et al., 2013), which can improve or strengthen the parent’s own emotional experience and attributions of their child (Sawrikar & Dadds, 2018). Positive parental attributions have been linked with robust child treatment outcomes and a strong parent-child relationship (Sawrikar et al., 2018). Consistent VEA also models prosocial communication that can be internalized into the child’s socioemotional skillset over time (Kochanska et al., 2013). Thus, VEA is an important component of warmth/affection that is essential to relationship building and children’s socioemotional learning. While the DPICS-IV captures some verbal endearments such as Unlabeled Praise (e.g., “*I love you*”), the VEA category of the WACS-I was largely designed to be orthogonal to DPICS-IV Praise categories and provides greater coverage of possible instances of warm parent verbalizations that otherwise go unaccounted for in the DPICS-IV. For example, a statement such as “*I had fun playing with you*” would be coded as VEA in the WACS-I, whereas the DPICS-IV would categorize this simply as Neutral Talk.

#### Tone/Pitch of Voice (TPV)

Another verbal cue linked with parental warmth, and central to affectionate communication and socioemotional development in children is modulated speech with positive vocal affect and heightened pitch (i.e., infant-directed speech or ‘parentese’) (Papoušek, 2007). According to Affection Exchange Theory (AET; Floyd, 2001; Floyd & Morman, 2001), affectionate communication is an adaptive trait that improves chances of survival and procreation. Researchers argue that humans have evolved to be able to differentiate vocal pitches given the indicator it provided regarding potential physical threat in their environment (e.g., smaller organisms such as babies and birds have higher modal pitches). In support, studies indicate that higher-pitched voices are perceived by humans to signal safety, affection, or friendliness, while lower-pitched voices can be encoded as indicating dominance or aggression (Floyd & Ray, 2003). Moreover, infants and young children show an innate preference for the rhythmic patterns associated with modulated and heightened tone or pitch because it assists them in becoming increasingly proficient in extracting contingencies in their environment (Papoušek, 2007). Over time, children learn to use their own vocal cues in an instrumental manner to communicate and have their needs met, which is critical to establishing a secure attachment (Stern, 1999). Thus, tone and pitch modulation (e.g., voice pitch increased to indicate excitement, speaking to child in a musical tone), hallmarks of parentese, are important for affectionate parent-child communication.

#### Facial Expressions (FE)

Facial expressions are also critical to affectionate communication and serve as important, non-verbal affiliative inputs that facilitate parent-child bonding (Feldman, 2012; Waller & Wagner, 2019). Facial expressions are a primary form of mutual communication between parent and child, particularly when children are still developing their verbal abilities (Maccoby, 1992). Facial expressions allow parents to understand and respond to their child’s needs, and mimicry and synchronicity between parent-child expressions facilitates greater attunement between both parties’ affective states (Tolleson et al., 2016). Exposing children to a wide emotional range via diverse facial expressions is an essential part of parental socialization that supports socioemotional and empathic development in children (Eisenberg et al., 2001). Consistent and varied use of facial expressions during interactions (e.g., expressions of excitement, enjoyment, and approval including smiling and laughing; surprise through raised eyebrows, widened eyes) provide children with ample opportunities to learn via modeling how to recognize and subsequently respond to others’ emotions—a critical process underpinning conscience development.

#### Physical Touch (PT)

Positive physical touch (e.g., hugging/caressing in humans, grooming/licking in most animals) is one of the most primitive and core non-verbal affiliative cues that signals safety and comfort. The seminal work of Harlow (1958) supports the notion that the need for physical touch (or “contact comfort”; p. 677) with a caregiver can overshadow even more primal, basic needs such as feeding. Specifically, he demonstrated that young rhesus monkeys who were exposed to a non-feeding, yet physically soft surrogate mother demonstrated more playfulness, exploration, and less stress than those with access to a feeding, yet cold wire surrogate mother. More recently, animal and human studies alike support the link between maternal care, in particular physical touch, and its ability to regulate stress responses and impact neuronal development and gene expression (Kommers et al., 2015). Beyond the psychophysiological level, the negative impacts associated with a lack of physical touch are also apparent at the attachment level. For example, infants who sought out, but ultimately failed to obtain bodily contact with their mothers, over time began displaying more behaviors consistent with an avoidant attachment (e.g., aversion to close bodily contact) (Ainsworth, 1979). Accordingly, it is hypothesized that positive physical touch (e.g., physical affection including kissing, ruffling/stroking child’s hair, putting arm around child) contributes to the foundational elements of warmth and attachment, with a lack of positive physical touch negatively impacting the parent-child dyad.

#### Reciprocal Play (RP)

A common non-verbal practice of highly warm parents is their ability to initiate, engage in, and sustain reciprocated play *with* their child. While imitative play, which is captured by DPICS-IV macro category, may involve parents engaging in ‘parallel play’ alongside their child (e.g., building own tower next to their child’s), initiating, and engaging the child in *ongoing, reciprocal* play (e.g., helping the child build their tower) provide three key benefits. First, reciprocated play highlights the parent as a present and attuned attachment figure who is attempting to meet the child’s socioemotional needs (Leclère et al., 2014; O’Neill et al., 2021), and provides an attention-rich interaction that is positively reinforcing for the child (Dadds & Hawes, 2006; Eyberg & Bussing, 2011). Second, parents engaging in *reciprocated* play also foster rich socioemotional learning opportunities that broaden their child’s behavioral repertoire. Through back-and-forth play, parents can model prosocial (e.g., turn-taking, negotiation) and emotionally responsive (e.g., sharing, helping) skills (Kochanska & Aksan, 2004). Third, reciprocal play whereby parent and child are *both active and interacting* participants can elicit mutual enjoyment, which establishes a positive feedback loop within the dyad. These positive interactions have a cumulative and buffering effect against prior or future aversive interactions (Niec, 2018). Together, reciprocal play (e.g., joining in on child’s games or imaginary play, playing peek-a-boo) serves as a vehicle through which children receive parental warmth, while strengthening their socioemotional skills and the overall parent-child relationship.

#### Eye contact

Similar to facial expressions, eye contact (or eye gaze) is one of the few sensory modalities used in early life to foster close relational bonds and establish attachment security. Indeed, eye contact with caregivers in early infancy lays the foundations for social cognition (Striano & Reid, 2006), and continues to underpin numerous relational processes (e.g., attachment style, emotion regulation, communicative processes) that impact the quality of the parent-child relationship (Dadds et al., 2011). Parents that initiate and sustain eye contact with their child are more likely to have warmer relationships characterized by attunement to their child’s emotions (Leclère et al., 2014). Conversely, rejecting or difficulties in sustaining eye gaze is associated with poorer parent-child relationship quality (Dadds et al., 2011). Additionally, eye gaze can provide important information that allows individuals to deduce the emotional state of others (Dadds et al., 2011), which in turn informs empathic responding (Skuse, 2003). Together these factors underscore the need to consider whether parents initiate and/or reject eye contact with their children when assessing warmth/affection given its strong social affiliative properties.

#### Body Posture

Body posture and physical orientation are known to correlate with affective states, and can be used alongside vocalizations (Zieber et al., 2013) and facial expressions (Meeren et al., 2005) to process emotional states. Despite this, few coding systems explicitly consider body posture when measuring parental warmth. Body posture generally varies along a spectrum, and a parent’s physical orientation and proximity to their child contributes to how open-to-closed their posture may be perceived. Open body postures (e.g., relaxed resting position, closeness to child, oriented to face the child) can promote a warm relationship by encouraging proximity seeking behavior if the child requires soothing while exploring their environment (Elfenbein et al., 2010). Thus, an open relative to closed body posture provides essential non-verbal cues to the child that communicates the parent’s presence, engagement, and availability for soothing; all of which strengthen attachment security and the parent-child relationship (Leclère et al., 2014).

### Phase 2: Coding System Refinement

Phase two involved developing a comprehensive coding manual in line with best practice (see Hawes et al., 2013), including clearly defined guidelines and behavioral indicators for each coding category (see Supplementary Information for manual). Despite the potential for social desirability bias, the same, in-clinic 5-minute child-led play situation from the DPICS-IV was retained to elicit warm parenting behaviors for three main reasons. First, using the child-led scenario helped to maintain some consistency with the current DPICS-IV system that is a core component of the PCIT protocol. Second, this child-led scenario allows for standardization across the assessment process, while also eliciting representative behaviors of interest (Heyman & Slep, 2004), both of which are essential to the development of a novel tool. Finally, given PCIT-CU is delivered in-clinic, it was reasonable to complete assessments of key outcomes, including parental warmth, under similar conditions.

#### Coding Micro Units

Since a hybrid coding approach was adopted from the outset, phase two involved deciding whether a micro or macro coding approach would be more appropriate to optimally measure each category. A micro coding approach was adopted for most of the parent warmth categories, whereby discrete units of behavior are tallied into specific categories on a moment-to-moment basis. This methodology parallels that of the DPICS-IV (i.e., tallying instances of ‘Do’ skills) and is useful for both researchers and clinicians who may want to examine changes in the frequency of a behavior over the course of treatment. Verbal affection and non-verbal affection categories were specified to be coded separately from one another to reduce demands placed on coder attention, enhance accuracy, and enable the calculation of individual and composite verbal and non-verbal scores. However, when used in tandem with the DPICS-IV, it is possible for parent verbalizations to fall under both a WACS-I and DPICS-IV category (e.g., an exclaimed “*I love you*” would be coded as UP in the DPICS-IV, and VEA, TPV and possible FE in the WACS-I). To support synchronicity across coding systems, it was decided that if a parent verbalization clearly fit across both DPICS-IV and WACS-I categories, DPICS-IV ‘Do’ skills would be prioritized, while WACS-I VEA codes would be prioritized over DPICS-IV Neutral Talk codes.

#### Coding Macro Units

Considering the established challenges of micro coding non-discrete units (e.g., eye contact, body posture) (Hawes et al., 2013), as well as to ensure the WACS-I more closely aligned to the DPICS-IV approach to coding broader constructs (e.g., enthusiasm, imitating the child), a macro coding approach was adopted for eye contact and body posture categories. Macro coding provided a relatively simple and time efficient method for assigning ratings and was especially appropriate given these behaviors are observed at a high frequency (Hawes et al., 2013). The framework for coding parent eye contact in the WACS-I was adapted from the ‘I-Love-You’ coding protocol (Dadds et al., 2012). Parent eye contact consisted of “initiate” (i.e., attempts to make direct eye contact with child) and “reject” (i.e., refusal to reciprocate attempted eye contact from child) eye contact items rated on a 5-point Likert scale (1 = *Never* to 5 = *Almost Always*). An overall parent eye contact total was computed by summing “initiate” eye contact scores with reverse coded “reject” scores. Parental body posture was also rated on 5-point Likert scale ranging from closed to open (1 = *closed/rejecting*, 3 = *neutral*, 5 = *open/accepting*).

#### Coding Team

Two expert coders (first and second author) separately coded *n* = 9 criterion videos of varying difficulty containing parent-child interactions using a draft WACS-I coding manual and initial inter-rater reliability was computed at 63%. Therefore, disagreements were reviewed together, and the manual was refined to include examples to enable more reliable coding of categories, with final expert coding established at 80% agreement between the same two expert coders. Nonetheless, it was possible that familiarity with the original *n =* 20 videos reviewed and the *n* = 9 specific videos used to establish criterion IRR may have artificially inflated the IRR between expert coders. Thus, after expert criterion IRR was established, a separate coding team was formed to complete training and coding with the finalized version of the WACS (1st edition; WACS-I) coding manual. Coders (*n* = 5 all female, trained masters-level or advanced undergraduate research assistants in psychology; *n* = 1 self-identified as Middle Eastern ethnicity, *n* = 2 as South-East Asian, *n =* 2 as Caucasian) underwent comprehensive training. This training included attending a 2.5-hour training workshop facilitated by two expert coders, and regular fortnightly to monthly coding meetings involving live practice coding, clarifications of coding rules, and monitoring of IRR. All coders were trained to reliability (i.e., minimum 80% agreement with expert coder on criterion videos; Hallgren, 2012) by an expert coder before commencing coding of a larger sample of videos available as part of a wider clinical trial (see Prasad et al., 2022 for details regarding psychometric properties of the WACS-I).

The WACS-I adopts a hybrid micro and macro coding approach and was intended to be used in tandem with the DPICS-IV to provide greater coverage of positive and warm parenting behaviours. Micro codes are calculated for how often caregivers: (i) vocally express affection (VEA), (ii) positively modulate their tone/pitch of voice (TPV), (iii) use animated facial expressions (FE), (iv) engage in positive physical touch (PT), and (v) reciprocal play (RP) with their child. Micro codes yield three sum scores: (1) Verbal WACS-I subtotal (VEA + TPV), (2) Non-verbal WACS-I subtotal (FE + PT + RP), and (3) WACS total score (sum of verbal and non-verbal WACS-I subtotals). The WACS also employs macro codes to compute a total parent eye contact score and parental body posture score. Expressions of parental warmth/affection may receive multiple codes at once (e.g., a parent smiling while hugging their child and exclaiming “*I am so lucky to have you*” would simultaneously qualify for FE, PT, TPV and VEA codes in the WACS-I). Future research will seek to define the ordering of WACS-I categories in terms of their importance to the parent-child relationship to develop a priority and decisions rule order, akin to the approach used in the DPICS-IV.

## Recommendations for Implementing WACS-I

### Protocols for High Quality Data

Using analogue interactions to induce behaviors of interest may be necessary and in turn, demand characteristics must be considered in the interpretation of obtained codes. Nonetheless, the fidelity of derived scores is also linked closely with the standardization of audio-visual recording and coding procedures. Observations used to develop the WACS-I formed part of a pre-assessment protocol (approximately 1.5–2 h long) that included clinical interviews with parents and laboratory child tasks. An important consideration was to conduct behavioral observations after clinical interviews to allow sufficient time for families to familiarize themselves with the clinic space and clinician. Instructions introducing and initiating the child-led play situation are standardized across observations and in accordance with DPICS-IV protocol. Further, key considerations for the set-up of audio-visual equipment (see Supplementary Information) included an unobstructed front-view of parent and child, and clear audio recording of their interaction with stitching of multiple camera angles, where possible. Similar to the DPICS-IV’s handling of inaudible parent verbalizations, WACS-I codes are not assigned when parent behavior cannot be adequately observed.

### Cultural Considerations

Given parenting and subsequent expressions of warmth/affection differ across cultures (Bornstein, 2012), it is imperative that the cultural background of parent-child dyads, as well as coders is taken into consideration. The WACS-I coding guidelines outline the importance of coded behaviors being sufficiently positive, affectionate, and appropriate to the context, however this can differ across cultures. For example, in some Eastern cultures parental affection may be characterized as instrumental support (e.g., financial sacrifices to afford quality education) rather than explicit, verbally expressed affection (Putnick et al., 2014). Similarly, the quality and intensity of parental to child eye contact may also differ across cultures (Bornstein, 2015). Further, valued child outcomes also differ cross-culturally and can impact observed parental warmth/affection behaviors. For example, while Caucasian mothers are more likely to intentionally foster assertiveness, and emotional expression, Asian mothers are more likely to promote emotional maturity and self-control (Bornstein, 2012). Thus, validating the WACS-I with ethnoracially diverse samples is critical, especially given the predominantly Caucasian sample reviewed in the development process. Such studies may help inform further revisions to the WACS-I that increase its cross-cultural relevance.

In line with prior studies (Melby et al., 2003), early coding meetings revealed that the cultural ethnicity of dyads and/or coders interacted and had the potential to influence ratings for ambiguous and even seemingly ‘cold’ parent behaviors. For example, a South Asian mother observed to redirect her child’s repeated bids for reciprocal play back to building a block tower was met with mixed reactions. Coders from Western cultural backgrounds perceived this behavior as “very harsh” or “extremely rejecting”. In contrast, coders from South-Asian and Middle Eastern backgrounds viewed this behavior as “not that atypical”, and “familiar” to their respective cultural contexts. Such differences in coder perception were resolved through team discussions that involved reviewing coding guidelines and joint practice coding of the non-Caucasian dyads available when possible. When implementing the WACS-I, we recommend: (i) proactively familiarizing the team with evidence-based factors related to the cultural context of raters and/or dyads that are known to bias coding, and (ii) regular coding meetings and joint coding practice with ethnoracially representative videos.

In conclusion, parental warmth is critical in supporting core developmental processes. The emergence of warmth-focused interventions underscored the need to develop a specialized coding system to comprehensively capture this multifaceted construct for clinical and research purposes. The WACS-I was developed to meet this need. Given the WACS-I was developed as a complementary coding system for PCIT-CU, future studies are critical to assessing whether WACS-I scores are cross-culturally relevant, internally reliable, coded consistently across coders, and correlate as expected with other criterion measures of parenting, and key child outcomes examined in PCIT-CU (see Prasad et al., 2022). These future directions can help clarify the mechanisms underpinning the development of antisocial behavior of children with CU traits and for refining warmth-focused treatments. Notably, the WACS-I and warmth-focused interventions may also hold clinical promise for other high-risk parents who stand to benefit from coaching around their warmth and reciprocity.

## Electronic Supplementary Material

Below is the link to the electronic supplementary material.

Supplementary Material 1
