# Supplementary material for: Conception and Development of the Warmth/Affection Coding System (WACS): A Novel Hybrid Behavioral Observational Tool for Assessing Parent-to-Child Warmth
Source: Res Child Adolesc Psychopathol. 2023 Apr 20;51(9):1357–69. doi: 10.1007/s10802-023-01055-y (PMC10474998; doi:10.1007/s10802-023-01055-y)
Supplement: Supplementary file 1 — Supplementary Material 1 [file 10802_2023_1055_MOESM1_ESM.docx]

**Supplementary Information**

**Warmth/Affection Coding System – 1^st^ Edition (WACS-I)**

**Coding Manual**

**Warmth/Affection Coding System – 1^st^ Edition (WACS-I)**

*Official Version 1.0*

*(May 2021)*

**Introduction**

The Warmth/Affection Coding System (WACS) is a hybrid behavioral observation coding system developed to capture positive verbal and non-verbal parental cues of warmth and affection expressed towards children during a dyadic interaction (i.e., 5-minute child-led free-play situation). The coding categories included in the WACS are empirically derived indicators of both verbal and non-verbal warmth and affection that are known to promote a strong and secure parent-child relationship.

This coding system is designed to be used in conjunction with the 5-minute child-led free-play situation (i.e., Child Directed Interaction) employed as part of the Dyadic Parent-Child Interaction Coding System – Fourth Edition (DPICS-IV; Eyberg, Nelson, Ginn, Bhuiyan, & Boggs, 2013) observation protocol. The current version of the WACS (WACS – 1^st^ Edition or WACS-I) was validated using the child directed interaction play scenario given this is a low-demand situation that allows parents to express warmth and affection with lowered chances for the occurrence of conflict or child non-compliance.

The WACS was designed to accompany the Parent-Child Interaction Therapy (PCIT) adaptation for children with Callous-Unemotional (CU) traits (PCIT-CU; Kimonis, Fleming, Briggs, Brouwer-French, Frick, Hawes, Bagner, Thomas, & Dadds, 2018). The coding categories outlined in this manual reflect various behavioral indicators and treatment targets that PCIT clinicians may wish to assess, track, and provide coaching around when administering PCIT-CU.

**WACS Categories**


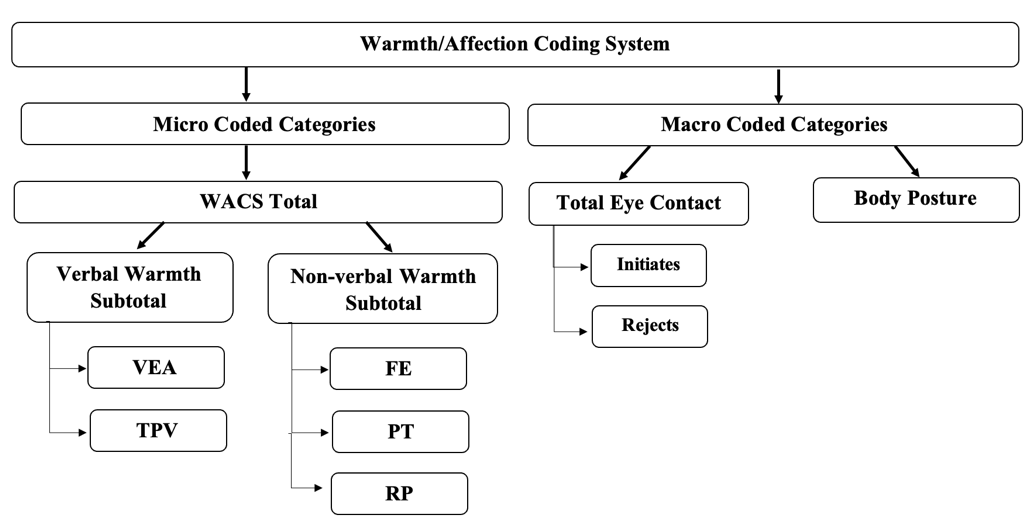
The current version of the WACS (WACS-I) contains a total of seven categories that capture parent-to-child warmth across both micro and macro coded scales (see Figure 1 for overview).

*Figure 1*. Overview of WACS Categories.

*Note.* WACS = Warmth/Affection Coding System; VEA= Vocally Expressed Affection; TPV = Tone/Pitch of Voice; FE = Facial Expression; PT = Physical Touch; RP = Reciprocal Play

**Micro coded categories**

Micro coded categories (Frequency counts during 5-minute child-led observation) are further split into *verbal warmth* and *non-verbal warmth* subcategories.

*Verbal Warmth*

- Modulation of Tone/Pitch of Voice (TPV)
- Vocally Expressed Affection (VEA)

*Non-verbal Warmth*

- Physical Touch (PT)
- Facial Expressions (FE)
- Reciprocal Play (RP)

**Macro (or global) coded categories**

Macro coded categories (5-point Likert rating scale) are used to assign an overall rating on the parent’s eye contact and body posture throughout the dyadic interaction.

*Eye contact*

- Rated on 5-point Likert scale (1 = *Never*, 5 = *Almost Always*)
  - Scale 1: Initiates / attempts to initiate eye contact
  - Scale 2: Rejects eye contact attempts by child (reverse coded)

*Body Posture*

- Rated on 5-point Likert scale (1 = *closed/rejecting*, 3 = *neutral*, 5= *open/inviting*)

**Calculating WACS scores**

There are 10 scores that can be calculated from the WACS (see below):

| **Micro coded** | **Categories** | **Calculation of score** |
| --- | --- | --- |
|  | Tone/Pitch of Voice (TPV) | Sum of total TPV frequency counts |
|  | Vocally Expressed Affection (VEA) | Sum of total VEA frequency counts |
|  | Verbal warmth Subtotal | Sum of total TPV + VEA frequency counts |
|  | Facial Expression (FE) | Sum of total FE frequency counts |
|  | Physical Touch (PT) | Sum of total PT frequency counts |
|  | Reciprocal Play (RP) | Sum of total RP frequency counts |
|  | Non-verbal Warmth Subtotal | Sum of total PT + FE + RP frequency counts |
|  | Total warmth | Sum of Verbal Warmth subtotal + Non-verbal Warmth subtotal |
| **Macro coded** | Total eye contact | Sum of ‘initiates/ attempts eye contact’ score (out of 5)  +  Sum of reverse scored ‘rejects child attempts of eye contact’ score (out of 5) |
|  | Body posture | Score on rating scale (out of 5) |

**Coding Procedure**

- If introducing the observation context to parent, use the following directions:
  - *5-minute warm-up section not coded*
    - “*In this situation, tell [child’s name] that he/she may play with whatever he/she chooses. Let him/her choose any activity he/she wishes. You just follow his/her lead and play along with him/her.”*
  - *After 5-minute warm-up period*
    - “*We’re halfway through this situation. Please continue to let [child’s name] lead the play.*”
- Coders are instructed to code clips twice to ensure that all verbal and non-verbal expressions of warmth are thoroughly captured.
- Round 1:
  - Code **verbal warmth micro categories only** (Tone/Pitch of Voice, Vocally Expressed Affection)
- Round 2:
  - First, code non-verbal warmth micro categories (Facial Expression, Physical Touch, Reciprocal Play)
  - Second, assign macro ratings for eye contact and body posture

**Notes for Coders**

- Displays of warmth and affection may result in micro codes for multiple categories
  - E.g., Parent smiles at their child while saying “*This was fun!*” = Facial Expression (Non-Verbal) + Vocally Expressed Affection (Verbal)
- Code all verbal and non-verbal warmth that is directed towards the child and/or objects/ products/ behaviors relating to the child. This can include warmth, affection and approval that is directed to the child, and/or toys they are, or were, playing with.
- Warmth may be coded in these instances even if the child is not directly paying attention to/oriented towards the parent.
- If coder is unsure whether a verbal/non-verbal cue is warm (i.e., not sufficiently positive), do not code. Ensure these ambiguous scenarios are noted in the appropriate log for review at the next coding meeting.

**Audiovisual Equipment Set-up**

- Ideally, two cameras in opposite corners of the room from one another should be used, with **at least one camera** directly facing the parent-child dyad within the designated play area.
- Cameras must have in-built audio recording capability. If possible, installing an overhead microphone may assist with improving the quality of recordings and subsequently accuracy of coding (especially for verbal warmth categories).
- See figure below for examples of audiovisual equipment set-up used for initial validation of the WACS-I.


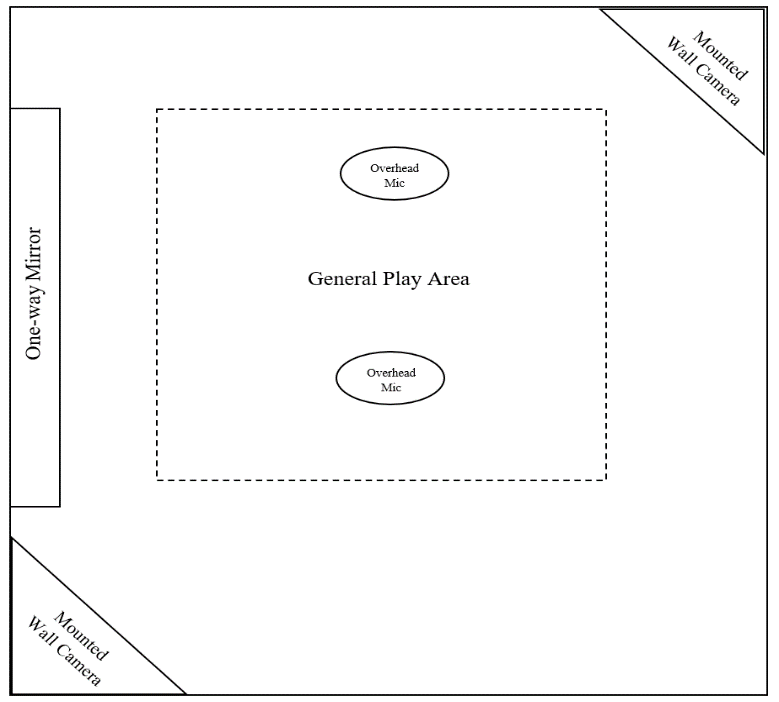


*Figure 2.* Room layout for parent-child observations.

**WACS-I**

**VERBAL WARMTH MICRO CODED CATEGORIES**

**Includes tone/pitch of voice (TPV) and vocally expressed affection (VEA)**

**TONE/PITCH OF VOICE (TPV)**

*Definition*

Tone/ Pitch of Voice is coded when a vocalization by the parent has a positive, rhythmic pattern of modulated tone and/or pitch of voice. This positive tone/pitch of voice should convey the parent’s interest, engagement, in the child’s attributes, activities, products or choices.

TPV is likely to be coded in context of infant-directed speech (also referred to as ‘parentese’ or ‘motherese’) which consists of long vowels, shorts consonants and a rhythmic pitch that is modulated according to context (see video for example: <https://www.youtube.com/watch?v=ZxmMfLBQOM8>).

*Notes*

- This category does not necessarily examine *what* parents are saying but rather *how* they are saying it.
- An increase in volume is not coded as TPV if it is not accompanied by an obvious and noticeable modulation of the tone and pitch.
- Modulation of TPV that occurs in the context of negative, hostile or critical statements are **not** coded.

*Examples*

- Infant-directed speech
- Musical/ sing-song tone/ modulated voice
- Enthusiastic/ animated speech
  - E.g., exclaiming, gasping, exaggerated emotional speech such as boisterous laughing
- Noticeably modulated pitch and tone that is appropriate to context
- Paced/slowed down speech
  - E.g., Repeating what the child said at a slowed rate, typically 1.5-2 second gap between phrases/words
    - Child: I want to pick up the pink car
    - Parent: “*You want to*…(1.5 sec) *locates car for child*…*pick up the pink car* (inflection)

*TPV Guidelines*

1. The change in tone/pitch of voice has to be:
   1. Noticeably different relative to the parent’s baseline TPV.
      1. It is recommended that coders assess the parents baseline tone/pitch of voice when speaking to the child *prior* to coding to serve as a point of reference.
   2. Sufficiently positive and in relation to the child’s attributes, activities, products or choices.
      1. Typical inflections at the end of questions may be coded as warmth *only* *if they are sufficiently positive within the context of the interaction.*
2. Tone/Pitch of Voice can be coded even if the parent verbalizations are delivered from the perspective of a toy/other object.
3. Changes to parent’s TPV are only coded separately if:
   1. Verbalizations are separated by a >2 second gap,

OR

- 1. There is no 2 second gap between verbalizations but there is a significant increase/decrease in TPV
     1. E.g., Parent: (in low pitch) “*I’m looking for Billy*… “ (1.5 sec) (in high pitch) “…*there he is!”*

1. If TPV is being coded in relation to a non-word, only code if it can be spelled
   1. E.g.,
      1. ‘*Ooooh*’ (with tone/pitch modulation)
      2. ‘*Mhmmmm’* (with tone/pitch modulation)
      3. ‘*'Aha!”* (with tone/pitch modulation)
2. TPV for parental laugh can be coded independent of whether parent’s facial expression changes or not. If parental laugh accompanied by facial expression, then code both TPV and FE.
   1. E.g.,
      1. *parent laughs in modulated way but keeps neutral face* 🡪 *Coded TPV*
      2. *parents laughs in modulated way while smiling widely* 🡪 *Coded TPV + FE*

**VOCALLY EXPRESSED AFFECTION (VEA)**

*Definition*

VEA is coded when there is a verbal expression of affection/ positive affirmation from the parent to child. The VEA category is intended to capture positively valanced statements that add to the overall warmth and quality of the interaction that are typically coded as ‘Neutral Talks’ in the DPICS-IV coding system.

*Notes*

- While praise (labeled and unlabeled) is not specified below in VEA criterion, using VEA alongside DPICS-IV Labeled and Unlabeled Praise categories can provide wide-ranging coverage of warm, positive parent verbalizations.

*VEA Examples and Guidelines*

1. VEA typically capture positive parent verbalizations that refer to:
   1. some **internal** attribute of the child (e.g., 'you are so smart' whereby smart cannot be observed)

OR

- 1. some **positive parental evaluation** of their own time/experience with the child (e.g., *‘This was so much fun!*')
     1. ***Note***: This can include references to retrospective events (e.g., ‘I had so much fun at the beach with you last week’).

OR

- 1. Some **positive** **parental evaluation** of a toy and/or fictional character’s retrospective, current or prospective experience in relation to the child
     1. *child making card for teddy bear at home* “*So thoughtful…Mr Bear is going to love the card!*”
     2. *while playing with Mr Potato Head* “*Whoa Mr Potato Head is having so much playing with you*!”

OR

- 1. Some **positive parental evaluation** of an object or activity the child is engaging with. Code VEA even if child is not explicit subject but it can be inferred from context that the parent is expressing approval of the child’s attributes, products or choices
     1. *child assembles farm animals in toy paddock* “*I like these animals…the sheep’s my favorite”*
     2. *child puts green shoes on Mr Potato Head* “*I like the green shoes. That’s pretty fancy!*”

1. VEA can include phrases that are not considered sufficiently positive to be praise in the DPICS-IV. Examples include:
   1. You are:
      1. so funny/ unique/ energetic/ persistent / one-of-a-kind/ concentrating/ interesting/ strong/ a hard worker
   2. You did that so:
      1. Quietly/ carefully/ thoughtfully
   3. Your ____ is:
      1. Colorful / fancy / unique / interesting
   4. Note: VEA should only be coded when intended to be positive (i.e., not in a sarcastic or mocking way).
2. VEA can include interpretations of child’s positive feelings about their attributes, products, or behavior (even if parental evaluation is not provided)
   1. *child smiles widely as parent hugs them* “*Aww you love big cuddles!*”
   2. *child jumps up and down at beginning of play time* “*Whoa, you seem excited when we play together”*
3. VEA can be coded when parent verbalization gives an account of their own behavior or desire if it conveys approval, availability, interest or adds to the overall quality of the interaction.
   1. Parent: “*I really want to draw with you”*
   2. Parent: “*I’m excited to see what we get to play today”*
   3. Parent: “*I missed having special play time with you*”
4. VEA should still be coded if it is accompanied with a NTA or DC/IC from the DPICS-IV coding system
   1. E.g., *'Darling*, stop it…' or *'Sweetie*, put the toy back.'
5. If parent sings to child, content of song may be coded for VEA for each complete phrase that expresses parental affection, care or approval of the child and/or their attributes, products or choices. E.g.,
   1. *parent singing ‘*You are my sunshine*’ to child*

“*You are my sunshine”*

🡪 Coded VEA

*“My only sunshine”*

🡪 Coded VEA

“*You make me happy…when skies are grey*”

🡪 Coded VEA

“*You’ll never know…how much I love you*”

🡪 Coded VEA

**WACS-I**

**NON-VERBAL WARMTH MICRO CODED CATEGORIES**

**Includes facial expression (FE), physical touch (PT), reciprocal play (RP)**

**FACIAL EXPRESSION (FE)**

*Definition*

Facial Expression is coded when the parent displays a facial expression towards the child’s attributes, products, activities or choices that is considered either: (a) sufficiently positive, and/or (b) conveys parental engagement and/or enthusiasm, or (c) is animated and appropriate to the context.

*Examples*

- **Animated or exaggerated facial expressions**
  - E.g., surprised face - raised eyebrows, widened eyes
  - E.g., happy face – widened eyes, smiling (may or may not include laughing
  - E.g., interested face – raised eyebrows, widened eyes, nodding head
  - E.g., acting/pretend play faces – animated expressions that are appropriate within the context of playing/engaging with the child
- **Any facial expression that is positive**
  - E.g., smiling, laughing, mirroring child’s expressions*
    - **Note*: mirroring of child’s expression is not coded if it is done in a way that is sarcastic, mocks or belittles the child.

*FE Guidelines*

1. Code facial expressions that are directed towards the child, even if the child is not always directly paying attention or orienting to the parent.
2. Different facial expressions are coded separately.
   1. *Note:* When followed in quick succession from one another, only code those expression that were displayed for 2 or more seconds.
3. If parent displays the same facial expression for a prolonged time – do not code multiple times. The next code should be when there is *a noticeable change in the parent’s prolonged facial expression.*
   1. E.g.,

Parent: *smiles* - Coded FE

Parent: *continues smiling for 5 seconds* – **Not** coded

Parent: *displays animated surprise face* - Coded FE

- 1. E.g.,

Parent: *smiles* - Coded FE

Parent: *returns to neutral face* - **Not** coded

Parent: *smiles again* - Coded FE

1. FE should only be coded if it is considered sufficiently and obviously positive and/or appropriate to the context. Examples include:
   1. If the child were to see the parent’s expression, the child should be able to easily tell that the parent’s expression is positive/ warm.
   2. If the parent displays a facial expression that is not positive/warm (e.g., sad/surprised/confused) it is appropriate within the context of playing/engaging with the child
      1. The following *would* be coded as FE

Child: *jumps from behind chair and roars to parent*

Parent: *displays surprised facial expression* - Coded FE

- 1. If the parent’s facial expression is not exaggerated but *noticeably* conveys genuine interest, engagement, approval and/or attention to the child (e.g., slightly raised eye-brows, good eye contact accompanied with nodding), FE is coded

*Note:* Observe the parent’s baseline facial expression *prior to coding* and use this as a reference point when deciding if facial expression is sufficiently positive and obvious. If unsure, do not code.

1. FE should only be coded if the expression is done in a positive/genuine way (e.g., not done sarcastically towards the child).
2. Facial expression should not be coded if the parent's face is not properly visible and discernible.

**PHYSICAL TOUCH (PT)**

*Definition*

Physical touch is any positive contact with the child that is initiated by the parent and conveys a desire for closeness/ engagement with child. This can include the parent touching the child with their body or with an object/toy.

*Examples*

- Hug
- Pat on head/ shoulder /leg /back
- Kiss / blowing kiss
- Tickling child
- Placing child on lap
- Ruffle or strokes child’s hair
- Adjusts child’s clothing/ hair / etc in loving way

Squeezes child’s cheeks

*PT Guidelines*

1. Physical touch coded if the positive action is done *to* the child. PT does not require reciprocation from the child.
2. For discontinuous touches that occur less than 2 seconds apart, coded as one PT. However, if 2 or more of the above examples are done simultaneously (e.g., hug + kiss) or sequentially (e.g., pats head then kisses), each physical touch is coded *separately* **only if** they are discernibly different behaviors.
3. Physical Touch may still be coded even if the child is not fully paying attention or does not initially respond positively out of surprise.
   1. *Note:* In these instances, please note in the coding log for review at coding meetings.

**RECRIPROCAL PLAY (RP)**

*Definition*

Reciprocal play is any positive physical activity enacted by the parent that is done in an attempt to initiate, sustain, engage with, or entertain the child and/or lead to reciprocated activity. This may also include instances in which a parent engages in an activity that has been chosen by their child and plays *with them* (not just alongside). The Reciprocal Play category captures greater parental effort (i.e., more sustained involvement required than physical touch), availability and/or interest in engaging in a reciprocal, back and forth interaction with the child.

*Examples*

- Peekaboo
- Dance with child
- Physical play (e.g., swing child around)
- Initiates hi-5
- Purposely do an action to make the child laugh
  - E.g., pretend to drop something, pretend to fall over
- Physical prompts for child while teaching (e.g., holding hand to write)
- Playing dolls with child and going along and/or adding to their storyline

*RP Guidelines*

1. Reciprocal play is coded only if the action is considered a sufficient attempt to engage the child and conveys the parent’s genuine interest/desire to interact with child.

E.g.,

Child: Mum come help me find the trains!

Parent: *loudly sighs and slowly gets up from chair* - Not coded RP

1. Reciprocal play is coded even if the child does not reciprocate or pay attention to parent’s efforts immediately.
2. Reciprocal play is coded if the parent attempts to use a toy/other prop to engage the child in some back and forth play/activity.
   1. E.g., Waving/playing with toy to gain child's attention/ engage in reciprocal play
3. If reciprocal play is not initiated with the child, but the parent opts-in to play with the child, code RP once initially as parent is attempting to *sustain* the play.
   1. E.g., During child-led play, parents are instructed to “Follow the child’s lead”. If the child chooses blocks, code RP for the initial buy-in when parent agrees to participate and sustain that chosen activity (e.g., starts helping child search for blocks).
   2. Only code subsequent RP if parent attempts to sufficiently:
      1. Engage the child
      2. Express interest in the shared activity
      3. Sustain the activity/interaction with the child
      4. Gain child’s attention with another activity

E.g..,

*Parent sees child playing with blocks*

Parent: *starts collecting blocks to assist child* - Coded RP

Parent: *collects blocks and begins stacking in silence without talking or engaging with child* - NOT coded RP

E.g.,

Child: *searching for Lego piece*

Parent: *helps take piece out of box and hands to child* - Coded RP

Parent: *continues searching, pulls out several pieces and gives to child* - Coded RP

E.g.,

Child: *drawing on paper at desk*

Parent: *sits down at desk* ‘I’m going to draw a rocket like you!’ – Coded RP

Parent: *sees child put down blue crayon after using* ‘I’m going to make mine blue like yours!’ * picks up blue crayon* - Coded RP

1. For prolonged actions, code RP once action has been completed before coding an additional RP. Only code subsequent RP’s for each **distinct attempt** to engage the child and/or initiate or sustain the play.
   1. E.g.,

Parent: *offers to search for red block from box, locates and gives to child* - Coded RP

Parent: *continues *passively* searching through box while child engages in their own building* - NOT coded

1. If 2 or more explicitly different actions are done simultaneously or sequentially, each action is coded *separately*.
   1. E.g.,

Parent: *hi-5’s child and passes them another block to continue building* - Coded RP x 2

1. Code RP if parent uses prompts (e.g., pointing to parts of toy) during reciprocated play as they are attempting to sustain/progress/ engage with the play.
2. It is recommended that coders wait until parent completes action before coding RP to reduce the likelihood of coding false positives.

E.g.,

Parent: *starts to pick up toy*

Coder: *codes RP* (incorrect)

Parent: *picks up toy to move out of way* - Not coded RP

1. Coders may use verbal and non-verbal information to guide coding for RP.
   1. E.g., Verbal Cue

Child: I want the blue block *starts to search*

Parent: I’m going to try and find the blue block… *searches and retrieves blue block for child* - Coded RP

E.g., Non-verbal cue

Child: *empties box of train sets and tracks*

Parent: *sits down and starts assembling tracks* - Coded RP

1. Code RP if parent mirrors and extends on child behavior (this may include play and non-play) if:
   1. The mirroring behavior is sufficiently obvious

OR

- 1. Parent verbalizes a statement that indicates their intention to mirror the child’s behavior and/or engage in a sustained interaction

E.g., Verbal indication

Child: *adjusts seating and crosses their legs and proceeds to draw*

Parent: “I’m going to cross my legs and start drawing just like you” *parent crosses legs and retrieves paper* – Coded RP

**WACS-I**

**MACRO CODED CATEGORIES**

**Includes eye contact and body posture**

**Parent Eye Contact**

The parent eye contact items and descriptors are drawn from the ‘I-Love-You’ Coding Protocol for a 90-second, structured task originally detailed by Dadds, Allen, Oliver, Faulkner, Legge, Moul, Woolgar, and Scott (2012), and later outlined in Dadds, Allen, McGregor, Woolgar, Viding, & Scott (2013). In this task, parents are instructed to *“…look [child’s name] in the eye and show him/her, in the way that feels most natural for you, that you love him/he*r” and trained researchers code ‘initiates/ attempts eye contact’ and ‘rejects child attempts at eye contact’ items on a 5-point Likert scale (1 = *Never*, 5 = *Almost Always*). A “Not Applicable” option was added for when the parent has not had an opportunity to initiate/attempt or reject/avoid eye contact.

***Scale 1: “Initiate or attempt eye contact with child”***

To what extent does the parent initiate, or try to initiate, eye contact with their child? This could be a verbal command such as, “Look at me” or any movement that is made to try to create eye contact including indirect eye contact (e.g., visually engaging with the other through the reflection in the mirror). The avoidance of eye contact would suggest a low score on this variable but not necessarily – a person may avoid eye contact initiated by others but initiate it themselves.

***Scale 2: “Reject/miss/ avoid eye contact attempt by child”***

To what extent does the parent reject their child’s attempt at eye contact? Rejection of eye contact might be physical, such as turning the head, putting hands in front of their face or shutting their eyes; or it could also be a verbal command such as, “Go away” or “Stop looking at me”. A low score on this variable would infer no difficulty in retaining eye contact with the other person.

**Body Posture**

Body posture is rated on a 5-point Likert scale ranging from negative/close to positive/open. When coding body posture, consider the parent’s orientation, proximity and wider body language.

On a scale of 1 to 5, rate the parent’s body posture toward the child.


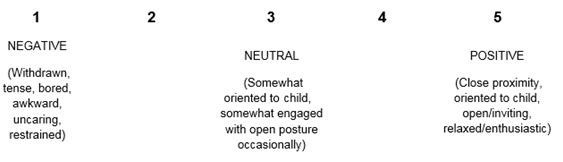


**Coding Sheet for WACS-I**

**
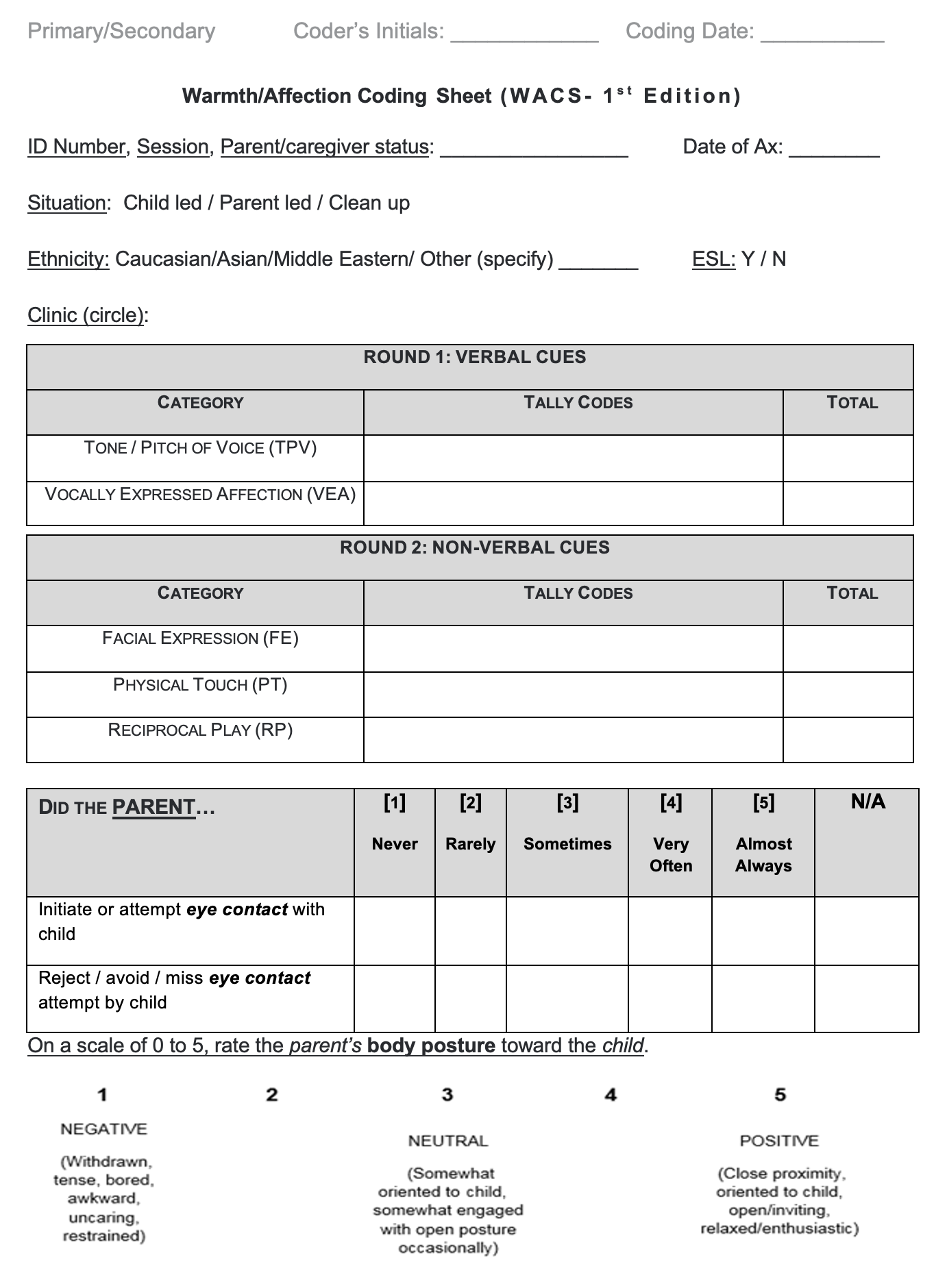
**
